# Traumatic iridial extrusion mimicking a conjunctival melanocytic neoplasm

**DOI:** 10.3332/ecancer.2016.620

**Published:** 2016-02-12

**Authors:** Pablo Zoroquiain, Maria SB Ganimi, Sarah Alghamdi, Julia V Burnier, Sultan S Aldrees, Miguel N Burnier

**Affiliations:** 1 Department of Pathology, Henry C Witelson Ocular Pathology Laboratory, McGill University, 1001 Boul Decarie, Montreal H4A 3J1, Canada; 2 Department of Pathology, School of Medicine, Pontificia Universidad Catolica de Chile, Marcoleta 377, Santiago 8330024, Chile; 3 Faculdade de Ciências Médicas e da Saúde de Juiz de Fora, Suprema, Alameda Salvaterra, 200 - Salvaterra, Juiz de Fora - MG 36033-003, Brazil; 4 Department of Ophthalmology, McGill University, 5252 Boul de Maisonneuve ouest, Montreal H4A 3S5, Canada; 5 Department of Ophthalmology, College of Medicine, King Saud University, PO Box 245, Riyadh 11411, Saudi Arabia

**Keywords:** conjunctival melanoma, iridial extrusion, ocular melanosis

## Abstract

Conjunctival melanoma is a rare malignant tumour of the eye. Its diagnosis represents a challenge for general pathologists due to low exposure to ocular biopsies and a broad differential diagnosis. In addition, conjunctival samples are often small and are associated with a high frequency of artefacts due to their processing. Here, we present the first case to date of a traumatic iridial extrusion masquerading as a conjunctival melanocytic neoplasm. An 83-year-old Asian man presented with a conjunctival-pigmented nodule surrounded by an area of diffuse pigmentation. Histopathology revealed in the nodule a well-demarcated lesion composed of spindle shaped melanocytes with thick-walled blood vessels. At higher magnification, the blood vessels were composed of thick walls with collagen fibres in an onion-skin-like arrangement. The histological findings were consistent with extruded iridial tissue. The map biopsies of the flat, pigmented lesion showed melanocytic cell proliferation with dendritic processes restricted to the lamina propria without any epithelial involvement, consistent with ocular melanocytosis. The diagnosis of conjunctival melanocytic lesions is challenging, and non-neoplastic conditions should always be included in the differential diagnosis. Pathologists should correlate clinicopathological findings and be familiar with the normal histology in order to achieve the correct diagnosis.

## Introduction

Conjunctival melanocytic neoplasms can be classified into benign neoplasms, such as nevi and primary acquired melanosis (PAM) without atypia; premalignant tumours such as primary acquired melanosis (PAM) with atypia, or malignant neoplasms such as conjunctival melanoma [1]. About 75% of conjunctival melanomas arise from primary acquired melanosis with atypia, 5% arise from nevi, while the remaining cases are de novo [2]. Furthermore, many conjunctival lesions can simulate a melanoma, including benign or malignant, melanocytic or non-melanocytic, and non-neoplastic entities known as pseudomelanosis.

As with other melanocytic lesions in different parts of the body, the diagnosis of conjunctival melanoma (CM) is a challenge. The main architectural features indicating a malignancy in a conjunctival melanocytic lesion are the same as those found in skin melanoma: asymmetry and the lack of maturation. However, the lack of an epithelial inclusion cyst within the melanoma is specific, but not sensitive, to conjunctival malignancy; in contrast, the presence of an epithelial cyst favours the diagnosis of a nevus. Cytological features are pleomorphism, the presence of nucleoli, and mitotic figures deep in the stroma. In addition, several non-melanocytic tumours are known to masquerade as conjunctival melanoma, which further obscures the diagnosis. Herein, we present a case of a traumatic iridial extrusion mimicking clinically a conjunctival melanoma and histopathologically a blue nevus.

## Case presentation

An 83-year-old Asian man was referred to the ophthalmology department by an optometrist for the evaluation of an asymptomatic pigmented lesion on the conjunctiva of the left eye. The patient’s medical history included ocular melanocytosis and ocular trauma that resolved spontaneously six months prior to presentation. On examination, a well-defined 10 × 5 mm pigmented nodule was seen on the superior medial conjunctiva of the left eye. The surrounding conjunctiva was also diffusely pigmented superficially. A clinical diagnosis of CM within an area of PAM was made. Excisional biopsy of the nodule and map biopsies from the flat-pigmented lesion were obtained in order to rule out any malignancy.

## Materials and methods

Haematoxylin and eosin (H & E)-stained sections from the nodule and map biopsies from the flat surrounding lesion were examined. Immunohistochemical studies in formalin-fixed-paraffin-embedded tissue were performed against HMB45, Melan-A, and Ki-67.

## Results

**Figure 1.**
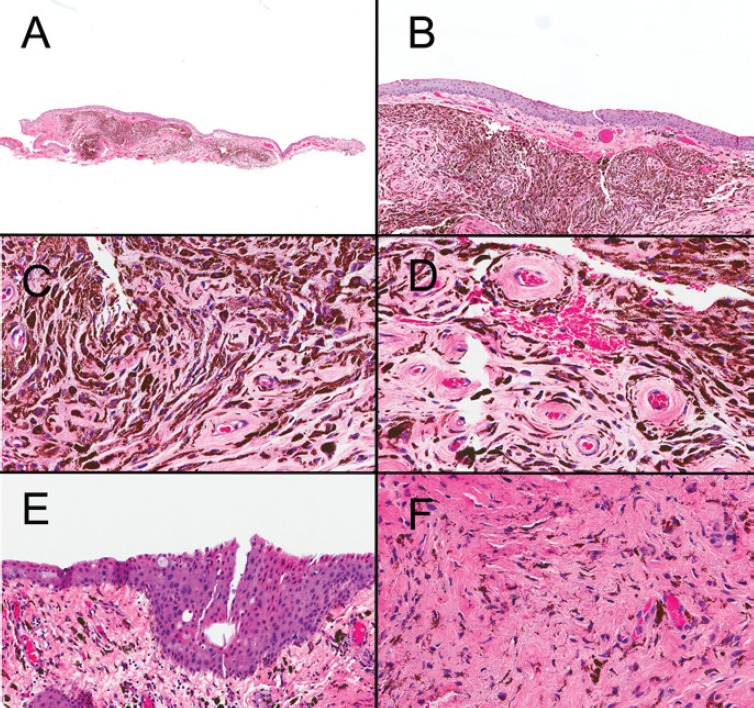
Conjunctival melanocytic nodule (A, B, C and D): (A) A well-circumscribed, non-necrotic, pigmented tumor is seen within the stroma (2×). (B) At higher power, a cleft between the tumor and the stroma is seen (10×). (C) Spindle-shaped melanocytes without dysplastic features are found in the stroma. (D) Collagen fibers with onion-skin-like configuration in thick-wall blood vessels are observed. Flat pigmented lesion adjacent to the nodule (E and F): (E) The flat, pigmented lesion adjacent to the nodule microscopically showed spindle-shaped melanocytes parallel to the surface epithelium. (F) At high magnification, no dysplastic changes were noticed. No atypical intraepithelial cells are seen.

An excisional biopsy from the nodule revealed a well-demarcated melanocytic lesion with thick-walled blood vessels (Figure 1A). In some areas, clefts could be observed between the lesion and the stroma (Figure 1B). At higher magnification, the lesion was composed of spindle-shaped melanocytes with dendritic prolongations and bland nuclear features (Figure 1C). The blood vessels accompanying the lesion had thick walls with collagen fibres in an onion-skin-like arrangement (Figure 1D). No mitotic figures or necrosis were seen. The melanocytic cells were positive for Melan-A and negative for HMB-45. The nuclear positivity index for the KI-67 antigen was less than 1%. Map biopsies of the flat, pigmented lesion showed benign melanocytes with dendritic processes, restricted to the lamina propria without any epithelial involvement (Figures 1E and 1F).

Based on the histopathological and immunohistochemical findings along with the clinical information obtained, the patient was diagnosed with extruded iridial tissue and ocular melanocytosis.

## Discussion

**Figure 2.**
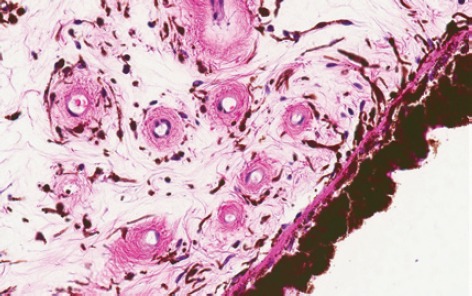
Iris from a human donor’s eye. Note the similarity with the lesion showed in Figure 1D. Onion-skin-like configuration of the collagen fibres can be seen in vessel wall.

It is important to be familiar with the peculiarities in ocular histology when ophthalmic specimens are being reviewed. Exposure to ocular histology is sometimes limited among general pathologists and dermatopathologists. In the present case, a biopsy was requested to rule out conjunctival melanoma; however, the histopathological picture was typical of an iridial tissue and guided us towards the correct diagnosis. The iris is characterised by the presence of dendritic melanocytes within a loose stroma, with surrounding characteristic thick-walled blood vessels (Figure 2). In this particular case, the presence of dendritic melanocytes running in parallel without epithelial involvement could have led to a misdiagnosis of blue nevus.

The differential diagnosis for a pigmented conjunctival lesion is broad, encompassing benign and malignant melanocytic proliferations, as well as non-melanocytic entities. Examples of benign melanocytic lesions are nevi (including blue nevus in this case), ocular melanosis, and PAM without atypia. Conjunctival nevi, similar to their skin counterpart, are circumscribed melanocytic proliferations in the epithelium or stroma of the conjunctiva and tend to occur in the interpalpebral area as yellow-brown nodules with associated epithelial pseudocysts [3]. Blue nevi, which were part of the differential diagnosis in this case, are composed of spindle and dendritic melanocytes that run parallel to the surrounding collagen fibres, respecting the overlying epithelium. They typically lack atypia and necrosis [4]. Ocular melanocytosis is a hamartomatous lesion in which pigmented melanocytes are found in the sclera rather than in the conjunctiva. Clinically, it is observed as a deeper melanosis that may affect the periorbital skin (oculodermal melanocytosis). This condition harbours an increased risk of developing a uveal melanoma, but not conjunctival melanoma [3, 5]. PAM, as the name implies, is an acquired condition seen in fair-skinned people. In cases of PAM without atypia, a benign lesion is characterised as either hyperplasia of melanocytes in the basal layer of the conjunctival epithelium (more than 1 melanocyte by 6 basal keratinocytes) or an increase in the amount of pigmentation and melanin transfer to the surroundings keratinocytes is seen [3]. However, in cases of PAM with atypia, a premalignant lesion, melanocytes show a degree of atypia as enlarged, hyperchromatic, and irregular nuclei. When these atypical cells remain in the basal layer, the condition is called PAM with low-grade atypia. However, PAM with high-grade atypia refers to when these cells show pagetoid spread [6]. Finally, if the neoplastic process infiltrates the stroma, it is referred to as conjunctival melanoma [7, 8].

**Figure 3.**
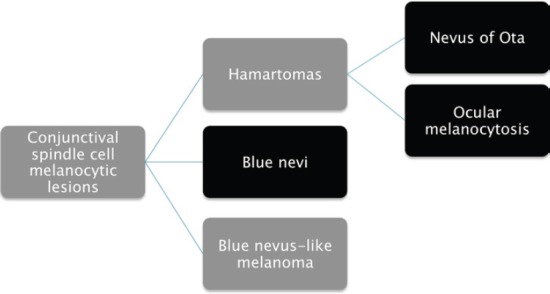
Differential diagnosis of spindle-shaped melanocytic lesions. Morphological features described in the entities in red overlaps.

In the map biopsies, the presence of spindle melanocytic cells led us to the diagnosis of spindle cell melanocytic lesion of the conjunctiva, as summarised in Figure 3. It is important to highlight that blue nevus and ocular/oculodermal melanocytosis share the same morphological picture. The main differences are that the former generally appears in adolescents and is clinically well demarcated, while the latter is congenital or arises in early childhood, being clinically diffuse in the entire conjunctiva and sometimes involving the skin (oculodermal melanocytosis) [9, 10]. For this reason, correct diagnosis of these tumours requires clinicopathological correlation.

There is a group of reactive non-neoplastic conditions, which are clinically referred to as pseudomelanosis and should be considered by the pathologist when reviewing a case with the clinical diagnosis of conjunctival melanocytic lesion. These include infections, foreign bodies, and chronic inflammation. Mycosis has been reported to mimic clinically conjunctival melanoma in a number of cases of dematiaceous fungi due to the presence of melanin in the wall of the fungal elements [11]. Foreign bodies have also been reported to clinically simulate conjunctival melanoma, including aluminium silicate material, mascara deposits, graphite from injury with a pencil, adrenochrome deposits from adrenalin analogues, and suture reactions [12–15]. Moreover, in our practice, we have encountered conjunctival chronic inflammation with siderophages clinically simulating a conjunctival extension from a primary uveal melanoma (data not published).

## Conclusion

The differential diagnosis for a pigmented conjunctival lesion is broad. In spindle melanocytic lesions of the conjunctiva, a multidisciplinary teamwork to obtain detailed medical history, perform clinical examination, and interpret tissue biopsies in light of the clinicopathological findings is essential to achieve the correct diagnosis. To the best of our knowledge, this is the first reported case of a traumatic iridial extrusion with associated ocular melanosis, clinically masquerading as conjunctival melanoma arising in a primary acquired melanosis.

## Conflict of interest

None.

## Disclaimer

This case was presented at the 55th International Academy of Pathology, October 2014 Bangkok, Thailand.
